# Changes in precarious employment in the United States: A longitudinal analysis

**DOI:** 10.5271/sjweh.3939

**Published:** 2021-03-31

**Authors:** Vanessa M Oddo, Castiel Chen Zhuang, Sarah B Andrea, Jerzy Eisenberg-Guyot, Trevor Peckham, Daniel Jacoby, Anjum Hajat

**Affiliations:** University of Illinois Chicago, Department of Kinesiology and Nutrition, Chicago IL, USA; University of Washington, Department of Economics, Seattle, WA, USA; University of Washington School of Public Health, Department of Epidemiology, Seattle, WA, USA; University of Washington School of Public Health, Department of Environmental and Occupational Health Sciences, Seattle, WA, USA; University of Washington Bothell, School of Interdisciplinary Arts & Sciences, Bothell, WA, USA

**Keywords:** employment condition, employment quality, health disparity, non-standard work, precarious work, precarity

## Abstract

**Objective:**

This longitudinal study aimed to measure precarious employment in the US using a multidimensional indicator.

**Methods:**

We used data from the National Longitudinal Survey of Youth (1988–2016) and the Occupational Information Network database to create a longitudinal precarious employment score (PES) among 7568 employed individuals over 18 waves (N=101 290 observations). We identified 13 survey indicators to operationalize 7 dimensions of precarious employment, which we included in our PES (range: 0–7, with 7 indicating the most precarious): material rewards, working-time arrangements, stability, workers’ rights, collective organization, interpersonal relations, and training. Using generalized estimating equations, we estimated the mean PES and changes over time in the PES overall and by race/ethnicity, gender, education, income, and region.

**Results:**

On average, the PES was 3.17 [standard deviation (SD) 1.19], and was higher among women (3.34, SD 1.20), people of color (Hispanics: 3.24, SD 1.23; non-Hispanic Blacks: 3.31, SD 1.23), those with less education (primary: 3.99, SD 1.07; high school: 3.43, SD 1.19), and with lower-incomes (3.84, SD 1.08), and those residing in the South (3.23, SD 1.17). From 1988 to 2016, the PES increased by 9% on average [0.29 points; 95% confidence interval (CI) 0.26–0.31]. While precarious employment increased over time across all subgroups, the increase was largest among males (0.35 points; 95% CI 0.33–0.39), higher-income (0.39 points; 95% CI 0.36–0.42) and college-educated (0.37 points; 95% CI 0.33–0.41) individuals.

**Conclusions:**

Long-term decreases in employment quality are widespread in the US. Women and those from racialized and less-educated populations remain disproportionately precariously employed; however, we observed large increases among men, college graduates and higher-income individuals.

Employment conditions in the US have drastically changed since the 1970s, due to changes in class relations, social policies, recessions, globalization, and technology (1, 2). The number of workers in high-quality, full-time employment, with adequate wages and benefits, has decreased over the last 40 years, while the number with low-quality, precarious employment (PE) has increased (2–7). Such trends have implications for myriad social and economic challenges facing society, including growing wealth and health inequities (7, 8). Researchers agree that multidimensional measures of PE can best capture trends in employment conditions (8–12). Yet, prior US estimates are unidimensional, cross-sectional, and/or rely on aggregated data, the latter of which may mask individual-level trends (2, 3, 13). Inadequate evidence about longitudinal, multidimensional trends in US employment conditions hinders our understanding of the causes and consequences of PE, as well as the development of effective policy interventions.

PE is best conceived as the accumulation of multiple unfavorable facets of employment quality (10). Although there is no single definition of PE, we build on a number of recent studies that have identified seven key dimensions of employment quality that capture PE’s multidimensionality: material rewards, working-time arrangements, employment stability, workers’ rights, collective organization, interpersonal relations, and training opportunities (10, 14–16). These dimensions represent the most widely used multidimensional operationalization of PE, drawn from a thorough review of the literature and expert consultations via the Employment Conditions Network (EMCONET) advisory group. Importantly, this conceptualization of PE captures both the contractual (eg, wages) and relational (eg, power relations) aspects of employment that play a critical role in worker health and well-being.

These seven dimensions are described in detail by Julia and colleagues (10). Briefly, material rewards denotes the wage and non-wage benefits afforded by an employment arrangement. Working-time arrangements denotes the length and intensity of working hours, underemployment, and schedule predictability. Employment stability captures continuity of employment, contractual temporariness and/or organizational changes (eg, downsizing), whereas the workers’ rights dimension denotes welfare state provisions associated with employment, such as access to health insurance or pensions. Collective organization refers to the possibilities (or lack thereof) for employee representation, most commonly operationalized though union representation. Interpersonal relations refers to the power of employees relative to management (eg, their ability to make decisions or control their schedule), and can include exposure to discrimination. Finally, training opportunities denotes workers’ opportunities for promotion or to enhance their skills. Prior studies employing these seven dimensions suggest that up to half of the European workforce experiences some precarity, including those in permanent full-time employment (15–22). However, most studies have employed cross-sectional data. Moreover, their estimates may not generalize to the US, given differences in labor markets and strength of social safety-nets (23).

Much of the empirical work in the US has narrowly focused on contract type alone (3). This approach is limited because it fails to capture the holistic experience of workers and the interrelation of dimensions, such as hours and wages (10). Notable exceptions include three US-based studies that have used multidimensional indicators. Peckham and colleagues employed a latent class analysis (LCA) approach to investigate the construct of employment quality using cross-sectional General Social Survey (GSS) data (24). Using similar indicators and the aforementioned seven dimensions, the authors identified eight employment quality types (16); five of these, constituting 58% of the sample, were deemed lower-quality or precarious (24). Cho and colleagues also applied an LCA approach to GSS data, although their PE indicators were subjective (eg, whether respondents described their benefits as “good”) rather than objective (eg, types of benefits respondents had access to) (25). These authors identified four employment types, two of which (40% of the sample), were deemed precarious. Finally, Eisenberg-Guyot and colleagues utilized the Panel Study of Income Dynamics and a sequence-analysis approach to identify mid-career employment quality trajectories based on dimensions similar to those employed in European-based analyses (26). They found heterogeneity between men and women with 46% of women and 20% of men in precarious or lower-quality employment trajectories (26). These studies improved upon prior US studies that employed unidimensional measures of precarity; however, they did not describe trends over time. Although Kalleberg’s seminal study on PE in the US did document time trends, he used national data to analyze separate trends for each indicator of PE, rather than analyzing individual-level data using a single multidimensional indicator (2).

Characterizing trends in PE using a multidimensional indicator is critical given that employment quality is increasingly recognized as a social determinant of health (8, 27). Scholars hypothesize three main mechanisms through which PE may be related to adverse physical and mental health (8). First, PE can cause deprivation (eg, insufficient income) and compromise access to necessities. Second, precariously employed individuals may have greater exposure to adverse physical (eg, toxic exposures) and psychosocial (eg, low control) working conditions. Third, precariously employed individuals may have limited control over both their professional and personal lives, leading to psychosocial stress. Importantly, poor employment quality may be contributing to widening health inequities, as women, people with lower education levels, and those from racialized groups tend to have a higher prevalence of PE (8, 13, 15, 17, 25–28).

To our knowledge, no prior studies have used a multidimensional indicator to estimate longitudinal trends of PE in the US. We addressed this gap using data from the National Longitudinal Study of Youth (NLSY) (1988–2016) and the Occupational Information Network (O*NET) database. The objectives of this study were to: (i) create a multidimensional continuous measure of PE in the US and (ii) describe changes in precarious employment over time, both overall and within subgroups. A better understanding of longitudinal trends is a critical first step for informing future policies aimed at improving PE and population health in the US.

## Methods

### Data sources

We used data from the 1988–2016 NLSY, which began collecting data in 1979 among a sample of 12 686 men and women (aged 14–21 years). The cohort’s profile and survey’s sampling methodology have been described elsewhere (29, 30). Briefly, the sample was designed to represent the civilian population of the US in 1979. In addition, this cohort oversampled Black, Hispanic and economically disadvantaged people. Individuals were interviewed annually through 1994 and biennially thereafter. About 55% (N=6912) of the original sample was retained in 2016. Respondents were consistently asked labor-market-related and demographic questions. To ascertain employment characteristics beyond those captured in NLSY, we linked individual-level NLSY data to occupation-level data from the O*NET database (2019 version 24.2). O*NET contains information on occupation-level characteristics for approximately 1000 occupations (31).

The Bureau of Labor Statistics permitted the use of the NLSY79 restricted-use data, which contains state-level geographic identifiers, and the University of Illinois Chicago Institutional Review Board deemed this secondary data analysis non-human-subjects research.

### Sample

Our original data set contained 12 686 individuals followed for an average of 8 waves between 1979 and 2016. We first restricted our analyses to 11 052 individuals who participated in at least two waves between 1988 (aged 23–31 years) and 2016 (aged 51–59 years). Then, to align with prior literature, we restricted to 10 281 individuals who were employed at least once (13, 16, 17, 20, 24, 25, 28). We further excluded individuals who had <40% of the survey items observed across their employed years combined (N=1730); this exclusion included self-employed individuals, who lacked data on many PE indicators. Finally, we excluded individuals with incomplete information on key demographic characteristics (eg, gender, race/ethnicity, education) (N=983). The final analytic sample contained 101 290 employed observations of 7568 individuals over an 18-wave or ~30-year period (7 waves annually from 1988–1994 and 11 waves biennially 1996–2016). Each individual contributed 13-waves of data, on average (interquartile range 7–17).

### Precarious employment score construction

We based our scale off European research conceptualizing PE as a multidimensional construct of accumulated adverse employment conditions and measured seven dimensions of the employment relationship, as described above: material rewards, working time arrangements, employment stability, workers’ rights and social protections, collective organization or empowerment, interpersonal relations, and training and employability opportunities (10–12).

### Variable selection and inclusion

Guided by prior studies on the topic (10, 15), alignment with the seven PE dimensions and data availability, we identified 13 survey indicators to operationalize our PE score (table 1). The promotion indicator was not available in 1989–1994; to partially address missingness, we carried the 1988 values (including missing values) forward to 1989–1993 and the 1996 values (including missing values) backward to 1994. In addition, we used single multivariate imputation to address missingness in the workers’ rights (9% missing), empowerment (10% missing), and opportunities (10% missing) dimensions using age, gender, race/ethnicity, marital status, region, education, occupation, industry, and auxiliary items within the same dimension (eg, using health insurance to impute for retirement plan). Combined, all other dimensions had less than 4% missingness.

**Table 1:** Description of the precarious employment (PE) dimensions and indicators used in the PE score.^a^ [pt=point.]

Dimensions	Item	Scoring rubric (Total ranges 0–7)
(1) Material rewards	(1.1) Relative wages ^b^	0.5 pt: total wages/salary in past year below state average in same year; otherwise 0
	(1.2) Paid vacation	0.5 pt: no paid vacation offered, 0 otherwise
(2) Work time arrangements	(2.1) Total hours	0.33 pt: total hours/week worked <20 or >40, 0 otherwise
	(2.2) Regular shift	0.33 pt: non-regular shift, 0 if regular day shift
	(2.3) Fixed hours	0.33 pt: varying hours, 0 if fixed hours
(3) Stability	(3.1) Weeks employed	0.5 pt: number of weeks worked/employed in past calendar year is <48 weeks, 0 otherwise
	(3.2) Tenure	0.5 pt: total tenure is <1 year with current employer, as of interview date, 0 otherwise
(4) Worker’s rights ^c^	(4.1) Health insurance	0.5 pt: health insurance not offered by employer, 0 otherwise
	(4.2) Retirement plan	0.5 pt: no retirement plan (other than social security) offered by employer, 0 otherwise
(5) Collective organization or empowerment ^c^	(5.1) Union membership	1 pt: wage not set by collective bargaining, or covered by union or employee contract, 0 otherwise
(6) Interpersonal relations ^c^	(6.1) Freedom^d^	1 pt: minimal freedom to make decisions without supervision (< median value), 0 otherwise
(7) Training and employability opportunities ^c^	(7.1) Promotion	0.5 pt: no promotion (or chance) since last interview, 0 otherwise
	(7.2) Training	0.5 pt: no on-the-job training, 0 otherwise

a The PES was created with observations from employed individuals only. Wages (1.1) and weeks employed (3.1) include information from all jobs (eg, total wages in the past year). All other indicators include information for one’s current or most recent job.

b State-year sample-specific estimate, which allowed our measure to incorporate the increases in income inequality over follow-up (i.e. as income inequality increased, a smaller proportion of the sample had wages above the sample mean).

c Missing data were imputed based on: age (continuous), gender (male, female), race/ethnicity (Hispanic, non-Hispanic Black, non-Hispanic White, non-Hispanic Other), marital status (single, married, separated, divorced, widowed), region (Northeast, Northcentral, South, and West), education (primary, secondary, undergraduate, graduate), occupation (e.g. managerial and professional) and industry (e.g. mining, utilities). For the workers’ rights and training dimensions, missing data were also imputed based on the reported items within the same domain.

d Data is from the O*NET database.

### Scoring

Consistent with previous research, we had no a priori rationale for upweighting any of the seven dimensions (13, 15). Therefore, each dimension was worth a maximum of 1 point, regardless of the number of variables in the dimension. For example, each of the three variables in the working time arrangements dimension was worth 0.33 points. To create the total PE score (PES) for each respondent in each year, we summed their scores for each of the dimensions, yielding a maximum score of 7, with 7 indicating the most precarious (range 0–7). We then adjusted the PES to age 30, using a linear regression of PES on age, age-squared, and year dummies, so that aging within our sample did not drive the observed time trends. To assess the prevalence of PE in various subgroups, we also created tertiles of PE pooled across all survey waves.

### Statistical analyses

Analyses were conducted in Stata/IC 14.2 (StataCorp LP, College Station, TX, US), incorporating NLSY survey weights and sample design parameters for descriptive statistics to account for clustered sampling, attrition, and oversampling (32).

Statistical analysis proceeded in three steps. First, we estimated the age-adjusted mean PES in the full sample, as well as by race/ethnicity, gender, educational attainment, income and region. Second, we estimated the proportion of respondents in each PES tertile overall and by sociodemographic characteristic. Finally, we used linear generalized estimating equation (GEE) models to quantify the overall change in the PES over time. We also examined heterogeneity in change over time within and between subgroups (race/ethnicity, gender, educational attainment, income, and region). We tested between-group differences in the change over time using an interaction term (eg, time × Hispanics). All GEE models included an unstructured correlation structure, categorical indicators of time (quartiles: 1988–1993, 1994–2000, 2002–2008, 2010–2016), and robust standard errors, clustered at the respondent level. For all GEE models, we used the Stata margins command to estimate the predicted average PES values for each time period and subgroup.

### Sensitivity analyses

We conducted several sensitivity checks. First, we used a data-driven principal component analysis (PCA) approach to construct the PES, which allowed us to assess the validity of our a priori choice of variable groupings and equally weighted domains. Second, to assess concordance between the theory-driven and data-driven scores, we computed percent agreement and Cohen’s Kappa across quartiles of the continuous measures. Third, we combined the materials rewards (relative wages, paid vacation) and workers’ rights (health insurance, retirement plan) dimensions to address ambiguity about whether health insurance belonged in the rights or material rewards dimensions (table 1). For example, Julia et al (10) classified “additional” insurance plans as a fringe benefit or material reward, but classified health insurance a workers’ right; however, nationalized healthcare is common in the European context, unlike in the US, where it is largely employer-sponsored. Fourth, we examined whether the estimated PES trends changed when including unemployed observations in our sample (N=22 154). In these analyses, we assigned unemployed individuals a PES of 7 (most precarious) since precariously employed individuals frequently move in and out of unemployment. Fifth, we used an external income cutoff – the age- and year-specific mean income as measured in the American Community Survey – to dichotomize people as higher- or lower-income, which ensured the cutoff was nationally-representative. In our primary analyses, we used the sample-specific state-year mean wage/income as a cutoff (see table 1 and supplementary material, www.sjweh.fi/show_abstract.php?abstract_id=3939, figure S1). Both approaches allowed our measure to incorporate the increases in income inequality over follow-up; as income inequality increased, a smaller proportion of the sample had wages above the sample mean (given the variables’ increasingly skewed distribution). Sixth, in our regression-based analyses, we tested whether including survey wave fixed-effects rather than categorical year indicators affected our estimates. Finally, we assessed the sensitivity of our results when using additional correlation structures (independent, exchangeable, auto-regressive).

## Results

### Descriptive statistics

Overall, the average age-adjusted PES was 3.17 [standard deviation (SD) 1.19]; the average PES was 2.96 (SD 1.23) in 1988, compared to 3.43 (SD 1.15) in 2016 (table 2, supplementary table S1). In the overall sample (ie, pooling data across years), tertiles of PE were defined as: low precarity: <2.62, medium precarity: 2.63–3.74, high precarity: >3.75. The proportion of respondents in high-precarity jobs increased from 27% in 1988 to 35% in 2016. Moreover, between 1988 and 2016, 31% of individuals went from low to medium or from medium to high precarity, with an additional 10% of the sample transitioning from low to high employment precarity over time.

**Table 2:** Average precarious employment score and prevalence of precarious employment, 1988–2016^a^ [CI=confidence interval; PE= precarious employment; PES=precarious employment score; SD=standard deviation]

Characteristics	N (% of sample)	Average PES (SD)	Prevalence of PE % (SD) ^b^
Full sample	101 290 (100)	3.17 (1.19)	31 (0.46)
Gender			
Male ^c^	52 572 (51.9)	3.02 (1.17)	26 (0.44)
Female	48 718 (48.1)	3.34 (1.20) ^d^	37 (0.48) ^d^
Race/ethnicity			
Non-hispanic white ^c^	52 852 (52.2)	3.14 (1.18)	30 (0.46)
Hispanic	19 023 (18.8)	3.24 (1.23) ^d^	34 (0.47) ^d^
Non-hispanic black	28 214 (27.9)	3.31 (1.23) ^d^	36 (0.48) ^d^
Non-hispanic other	1201 (1.2)	3.26 (1.24) ^d^	35 (0.48) ^d^
Educational attainment			
Primary school ^c^	499 (0.5)	3.99 (1.07)	60 (0.49)
High school	50 195 (49.6)	3.43 (1.19) ^d^	40 (0.49) ^d^
College	39 546 (39.0)	3.03 (1.16) ^d^	26 (0.44) ^d^
Graduate	11 050 (10.9)	2.65 (1.05) ^d^	15 (0.36) ^d^
Wages/salary			
Below state median ^c^	50 650 (50.0)	3.84 (1.08)	53 (0.50)
Above state median	50 640 (50.0)	2.60 (0.97) ^d^	12 (0.33) ^d^
Region			
South ^c^	40 601 (40.1)	3.23 (1.17)	32 (0.47)
Northeast	16 112 (15.9)	3.03 (1.17) ^d^	27 (0.44) ^d^
North Central	24 368 (24.1)	3.17 (1.20) ^d^	31 (0.46) ^d^
West	20 209 (20.0)	3.18 (1.25) ^d^	32 (0.47)

a Estimates are adjusted to age 30 and are weighted using the NLSY custom weights for the whole study period (1988-2016).

b Represents the highest tertile of precarious employment. Tertiles were generated based on the pooled sample across all years (N=101,290 observations).

c Reference group for statistical testing. We used t-tests (average PES) and chi-squared tests (prevalence of PE) to test for differences between the reference and other group.

d P<0.05

The average age-adjusted PES and the prevalence of PE were significantly higher among women (3.34, SD 1.20), people of color (Hispanics: 3.24, SD 1.23; non-Hispanic Blacks: 3.31, SD 1.23), those with less education (primary: 3.99, SD 1.07; high school: 3.43, SD 1.19), those with lower-incomes (3.84, SD 1.08), and those residing in the South (3.23; SD 1.17).

### Time trends in precarious employment

**Figure 1:**
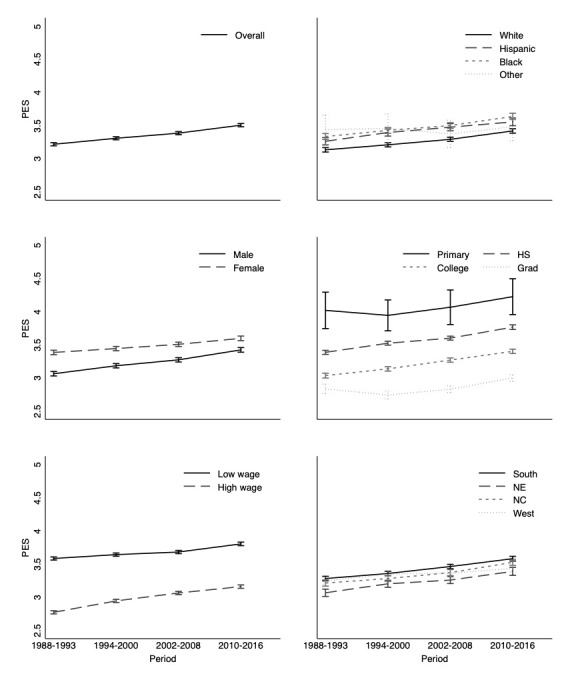
Precarious employment score over time, 1988-2016.^a, b, c^ [CI=confidence interval; HS=high school; NC=north central; NE=north east; PES=precarious employment score.] ^a^ We estimated the PES using a GEE model and the categorical indicators of time periods. We then predicted the average PES at each time period with their 95%CI. ^b^ Estimates are adjusted to age 30 years. ^c^ Low wage is defined as below the state-year median, high wage is defined as above the state-year median.

From 1988 to 2016, the overall PES increased by 0.29 points [95% confidence interval (CI) 0.26–0.31) or by 9% from 3.22 (95% CI 3.20–3.24) to 3.51 (95% CI 3.48–3.53) (table 3, figure 1). Except for the interpersonal relations dimension, all dimensions showed increasing precarity over time (data not shown).

**Table 3:** Time trend of precarious employment score (PES) overall and by subgroups.^a^ [CI=confidence interval; NH=non-Hispanic; TP=time period.]

	Average PES (95% CI)	Difference (95% CI) ^b^	Time × subgroup (95% CI) ^c^
Overall time trend			
1988–1993 (TP1)	3.22 (3.20–3.24)	Reference	
1994–2000 (TP2)	3.31 (3.29–3.33)	0.09 (0.07–0.11)	
2002–2008 (TP3)	3.39 (3.37–3.41)	0.17 (0.15–0.19)	
2010–2016 (TP4)	3.51 (3.48–3.53)	0.29 (0.26–0.31)	
Time trend by race/ethnicity			
TP1 NH White	3.13 (3.10–3.17)	Reference	
TP2 NH White	3.21 (3.18–3.24)	0.08 (0.05–0.10)	
TP3 NH White	3.29 (3.26–3.32)	0.16 (0.13–0.19)	
TP4 NH White	3.42 (3.39–3.45)	0.28 (0.25–0.32)	
TP1 Hispanic	3.26 (3.21–3.32)	Reference	
TP2 Hispanic	3.40 (3.34–3.45)	0.13 (0.09–0.17)	0.06 (0.01–0.10)
TP3 Hispanic	3.48 (3.42–3.53)	0.21 (0.16–0.26)	0.05 (-0.01–0.11)
TP4 Hispanic	3.56 (3.50–3.61)	0.29 (0.24–0.35)	0.01 (-0.06–0.08)
TP1 NH Black	3.33 (3.29–3.38)	Reference	
TP2 NH Black	3.43 (3.38–3.47)	0.09 (0.06–0.13)	0.02 (-0.03–0.06)
TP3 NH Black	3.50 (3.46–3.54)	0.17 (0.12–0.21)	0.01 (-0.05–0.06)
TP4 NH Black	3.64 (3.59–3.68)	0.30 (0.25–0.35)	0.02 (-0.05–0.08)
TP1 NH Others	3.44 (3.22–3.66)	Reference	
TP2 NH Others	3.46 (3.24–3.67)	0.02 (-0.13–0.17)	-0.06 (-0.21–0.10)
TP3 NH Others	3.37 (3.17–3.58)	-0.06 (-0.24–0.11)	-0.22 (-0.40–-0.05)
TP4 NH Others	3.48 (3.27–3.69)	0.04 (-0.14–0.23)	-0.24 (-0.43–-0.05)
Time trend by gender			
TP1 Male	3.06 (3.03–3.10)	Reference	
TP2 Male	3.18 (3.15–3.22)	0.12 (0.10–0.15)	
TP3 Male	3.27 (3.24–3.31)	0.21 (0.18–0.24)	
TP4 Male	3.42 (3.39–3.46)	0.35 (0.33–0.39)	
TP1 Female	3.38 (3.35–3.42)	Reference	
TP2 Female	3.44 (3.41–3.47)	0.06 (0.03–0.09)	-0.06 (-0.10–-0.02)
TP3 Female	3.51 (3.47–3.54)	0.12 (0.09–0.16)	-0.09 (-0.13–-0.04)
TP4 Female	3.60 (3.56–3.63)	0.21 (0.18–0.25)	-0.15 (-0.20–-0.10)
Time trend by education level			
TP1 Primary School	4.02 (3.74–4.29)	Reference	
TP2 Primary School	3.94 (3.71–4.18)	-0.08 (-0.29–0.14)	
TP3 Primary School	4.07 (3.81–4.33)	0.05 (-0.25–0.35)	
TP4 Primary School	4.23 (3.96–4.50)	0.21 (-0.16–0.57)	
TP1 High School	3.39 (3.36–3.42)	Reference	
TP2 High School	3.52 (3.49–3.55)	0.14 (0.11–0.16)	0.21 (-0.00–0.43)
TP3 High School	3.60 (3.57–3.63)	0.21 (0.18–0.25)	0.17 (-0.13–0.47)
TP4 High School	3.77 (3.73–3.80)	0.38 (0.34–0.42)	0.17 (-0.19–0.54)
TP1 College	3.03 (3.00–3.07)	Reference	
TP2 College	3.14 (3.10–3.17)	0.10 (0.07–0.13)	0.18 (-0.04–0.39)
TP3 College	3.27 (3.24–3.30)	0.23 (0.20–0.27)	0.19 (-0.11–0.49)
TP4 College	3.40 (3.37–3.43)	0.37 (0.33–0.41)	0.16 (-0.21–0.52)
TP1 Graduate	2.84 (2.76–2.91)	Reference	
TP2 Graduate	2.74 (2.68–2.80)	-0.09 (-0.15–-0.03)	-0.02 (-0.24–0.20)
TP3 Graduate	2.83 (2.78–2.88)	-0.01 (-0.09– 0.07)	-0.05 (-0.36–0.26)
TP4 Graduate	3.00 (2.95–3.05)	0.17 ( 0.09– 0.24)	-0.04 (-0.41–0.33)
Time trends by income			
TP1 Below Median	3.58 (3.56–3.61)	Reference	
TP2 Below Median	3.64 (3.61–3.67)	0.06 (0.03–0.08)	
TP3 Below Median	3.68 (3.66–3.71)	0.10 (0.07–0.13)	
TP4 Below Median	3.80 (3.76–3.83)	0.22 (0.19–0.25)	
TP1 Above Median	2.77 (2.75–2.79)	Reference	
TP2 Above Median	2.94 (2.92–2.97)	0.17 (0.15–0.19)	0.11 (0.08–0.15)
TP3 Above Median	3.06 (3.04–3.09)	0.29 (0.27–0.32)	0.19 (0.16–0.23)
TP4 Above Median	3.16 (3.13–3.18)	0.39 (0.36–0.42)	0.17 (0.13–0.21)
Time trends by region			
TP1 South	3.28 (3.25–3.21)	Reference	
TP2 South	3.36 (3.32–3.39)	0.08 (0.05–0.11)	
TP3 South	3.46 (3.43–3.49)	0.18 (0.15–0.22)	
TP4 South	3.58 (3.54–3.62)	0.30 (0.26–0.34)	
TP1 Northeast	3.07 (3.01–3.12)	Reference	
TP2 Northeast	3.20 (3.15–3.25)	0.14 (0.09–0.18)	0.06 (0.01–0.11)
TP3 Northeast	3.26 (3.20–3.31)	0.19 (0.14–0.25)	0.01 (-0.05–0.08)
TP4 Northeast	3.39 (3.33–3.45)	0.32 (0.26–0.39)	0.02 (-0.05–0.10)
TP1 North Central	3.21 (3.17–3.26)	Reference	
TP2 North Central	3.28 (3.23–3.32)	0.07 (0.03–0.11)	-0.00 (-0.06–0.04)
TP3 North Central	3.37 (3.32–3.42)	0.16 (0.11–0.20)	-0.02 (-0.08–0.03)
TP4 North Central	3.52 (3.48–3.57)	0.31 (0.26–0.36)	0.01 (-0.05–0.07)
TP1 West	3.24 (3.19–3.29)	Reference	
TP2 West	3.35 (3.30–3.40)	0.10 (0.06–0.15)	0.03 (-0.02– 0.08)
TP3 West	3.36 (3.31–3.41)	0.12 (0.07–0.17)	-0.06 (-0.13–-0.00)
TP4 West	3.43 (3.38–3.49)	0.19 (0.13–0.25)	-0.11 (-0.18–-0.04)

a The PES is adjusted to age 30. Estimates were calculated using 6 separate GEE regression models with an unstructured correlation structure. All models included categorical indicators of year (TP: 1988–1993, 1994–2000, 2002– 2008, 2010–2016) and employed robust standard errors. Subgroup models include a TP × subgroup interaction term.

b Compares within subgroup change over time. For example, it compares the PES for NH Whites in TP 1 to the PES for NH Whites in TP 2.

c Compares the between subgroup change over time. For example, the change in PES between TP 1 and TP 2, comparing NH Whites (0.08) and Hispanics (0.13), is 0.06.

Between 1988–1993 and 2010–2016, the PES increased by about 9% among non-Hispanic Whites (difference: 0.28 points; 95% CI 0.25–0.32), Hispanics (difference: 0.29 points; 95% CI 0.24–0.35); and non-Hispanic Blacks (difference: 0.30 points; 95% CI 0.25–0.35). However, the increase observed among non-Hispanics Whites did not significantly differ from the increase among Hispanics (interaction: 0.01 points; 95% CI -0.06–0.08) or non-Hispanic Blacks (interaction: 0.02 points; 95% CI -0.05–0.08). Meanwhile, employment quality improved among people of “Other” races relative to the change among non-Hispanic Whites (interaction: -0.24 points; 95% CI -0.43– -0.05). The 11% increase observed among males (difference: 0.35 points; 95% CI 0.33–0.39) was significantly larger than the 6% increase among females (difference: 0.21 points; 95% CI 0.18–0.25) (interaction: 0.15 points; 95% CI 0.20–0.10).

The PES remained persistently high among the lowest-education subgroup over follow-up, but the precision was poor and the increase over time was not statistically significant (difference: 0.21 points; 95% CI -0.16–0.57). The PES significantly increased among all other education subgroups; we observed an 11% (0.38 point; 95% CI 0.34–0.42), 12% (0.37 points; 95% CI 0.33–0.41), and 6% (0.17 points; 95% CI 0.09–0.24) increase in the PES among those with a high school, college, and graduate level of education, respectively. Employment precariousness worsened over time among both lower- (difference: 0.22 points; 95% CI 0.19–0.25) and higher-income (difference: 0.39 points; 95% CI 0.36–0.42) individuals; however, workers with higher wages experienced a 14% increase in PE over time, compared to 6% among workers with lower wages (interaction: 0.17 points; 95% CI 0.13–0.21).

The score increased by about 9% (0.30 points) within most regions. Compared to the South, the change over time was only different in the West (interaction: -0.11 points; 95% CI -0.18– -0.04).

### Sensitivity analyses

Overall, the score was robust to our sensitivity checks. First, although the variables grouped in our PCA approach differed from those grouped in our theoretical approach (supplementary figure S2, panel A), the qualitative trend in the scores was similar between 1988 and 2016 and there was evidence for the two scores’ agreement (supplementary table S2). Second, when we combined the material rewards and workers’ rights domains, trends were similar to our primary specification (supplementary figure S2, panel B). Third, as in our primary sample, we observed increases in employment precarity over time when we included the unemployed observations of the same respondents; however, the increases were larger in magnitude (supplementary figure S2, panel C, table S3). For example, when including the unemployed, the overall PES increased by ~13% (difference: 0.50 points; 95% CI 0.46–0.54), including by 12% among non-Hispanic Whites (difference: 0.44 points; 95% CI 0.38–0.49), 14% among Hispanics (difference: 0.54 points; 95% CI 0.44–0.63), and 15% among non-Hispanic Blacks (difference: 0.60 points; 95% CI 0.52–0.67). Additionally, the increase was larger among non-Hispanic Blacks (interaction: 0.16; 95% CI 0.06–0.25) compared to non-Hispanic Whites. Overall trends were also similar when we used an alternative definition of lower- and higher-income (supplementary figure S2, panel D, table S4). When we used year fixed-effects rather than categorical indicators, results were larger in magnitude; there was a 17% increase in the PES comparing 1988 to 2016 (difference: 0.52 points; 95% CI 0.48–0.56) (supplementary figure S3, tables S5 and S6). Finally, results were similar in magnitude and significance when employing independent, exchangeable, and auto-regressive correlation structures (data not shown).

## Discussion

To our knowledge, this is the first US-based study to use longitudinal, individual-level data, and a multidimensional indicator to describe employment precariousness over time. We also measured PE on a continuum, which we believe allowed for a more nuanced approach to assessing employment quality than the binary indicators predominately used in prior research. We found that the average PES throughout follow-up was significantly higher among people of color, women, people with lower levels of education, and people with lower income. Moreover, between 1988 and 2016, the overall PES significantly increased indicating worsening employment quality over time. Finally, we observed the largest increases in PE among males, people with a college education and higher-income individuals, suggesting long-term decreases in employment quality are widespread in the US, rather than confined to marginalized segments of the labor market.

Prior US-based trend estimates are based on unidimensional indicators and/or repeated-cross-sectional data, making comparisons with the present study challenging. However, our finding that PE increased in the US over the last 30-years is generally consistent with repeated-cross-sectional data from the GSS, which suggested that non-standard work arrangements (temporary, on-call, contract workers) increased by about 11% from 2006 to 2010 (3). Our findings are also consistent with Kalleberg, who reported an increase in PE between 1970 and 2002, characterized by increased workloads, time pressures, hours worked, and insecurity (2).

Based on prior literature documenting differential opportunities for workers across the labor market due to factors like discrimination and sexism, we hypothesized that people of color, women, and individuals with lower levels of income and education would experience the largest increases in PE (13, 15, 17, 25, 26, 28). Women may face employment-related discrimination and are often responsible for childcare, which may keep them less attached to the labor force (27, 33). People of color are more likely than White people to experience job insecurity, leave their jobs involuntarily, and face employment-related discrimination (34–37). Meanwhile, higher education levels may afford workers more favorable employment conditions, like autonomy, stability and opportunities for advancement (27). Consistent with prior studies, we also found that PE was persistently higher and increased over follow-up among women and those from racialized and less-educated populations (13, 15, 17, 25, 26, 28). However, contrary to our hypothesis, we observed the largest change over time in precarity among males and college-educated and higher-income individuals. There are a number of plausible explanations for this finding. Larger increases for the most advantaged groups could, in part, reflect the fact that their PES was lower in 1988 and thus, there was a greater opportunity for their employment quality to worsen. The relatively large increase in PE among males may also be due to the declining rate of union membership in the US, as union membership is associated with better employment quality and historically, was more common among males (38). For example, the proportion of male workers covered by labor union contracts decreased from 25% to 11% from 1983 to 2018, whereas women’s union participation only decreased from just 15% to 10% over that time period (39). The increasing precarity among those with college degrees could reflect increasing educational attainment in the US; college degrees may no longer afford workers additional bargaining power and prestige as they have become more common.

More broadly, large increases in PE over time among males and those with higher-educations or incomes suggests that PE is a more structural phenomenon, affecting large segments of the population. Our findings are generally consistent with scholars’ hypothesis that employment quality in the US has declined even for individuals employed in “good”, permanent, or Standard Employment Relationship (ie, permanent, full-time, regularly scheduled work with secure wages) jobs (2, 13, 17, 39). Kalleberg reports that overall, employment has gotten harder, more insecure, and largely lacks benefits or opportunities for advancement (2). Nonetheless, our findings do contrast with several US Bureau of Labor Statistics studies that have examined only contract type and found a relatively stable prevalence of non-standard employment arrangements (3, 13). However, we believe these studies are limited, as contract type is only one dimension of employment that impacts worker well-being. Moreover, recent evidence suggests such studies may be undercounting emerging forms of precarious work (eg, gig employment) (7, 40, 41).

Importantly, our findings may have implications for worker health and well-being. For example, material deprivation, occupational hazards, and stressful environments, could prevent people from obtaining necessities (eg, health care and housing), limit health-promotion (eg, leisure time physical activity), and increase psychological distress (eg, anxiety, depression) (8). Thus, this widespread increase in PE could harm public health in the US. Moreover, the persistently greater level of PE among marginalized populations may contribute to health disparities. Furthermore, in our specification that included the unemployed in our sample, there was a greater increase in PE among non-Hispanic Blacks and Hispanics than among non-Hispanic Whites, suggesting that changes in PE may exacerbate racial/ethnic health disparities.

### Strengths and limitations

Key strengths of this study included our use of a multidimensional, longitudinal measure of PE, which allowed us to describe employment precariousness over time and among subgroups in the US. We conducted our analyses over a 30-year time-period, using more than 100 000 observations and conducted many sensitivity checks, which supported our overall inferences. Although some scholars suggest that PE is best measured from surveys that are developed specifically to capture the health relevant aspects of precarity, we also demonstrated the utility of using secondary data to longitudinally measure PE in the US with a multidimensional indicator (10).

Nevertheless, our study had limitations. First, the NLSY does not sample with replacement and in any given wave the age distribution was narrow (seven years). Although the score was age-adjusted, the trends over time may be most generalizable to the 1957–1964 birth cohort sampled. The labor force is now more diverse and with the worsening employment quality over time, it is possible that younger generations are experiencing more precariousness earlier in their careers. Because we have seen growth in both low- and highly precarious jobs, younger generations’ potential for improvement in employment quality may be more limited over time (ie, they may have a more difficult time closing the gap). Thus, PE may be contributing to increasing inequality. Second, after 2008, wages in our sample grew faster than the national average (data not shown). This may be because workers in our sample were older than the national average after 2008 (42), and because more-precarious workers were more likely to drop out of the sample during follow-up. Thus, we may have underestimated the extent to which PE increased over time. Nonetheless, we used age-adjustment and sampling weights, as appropriate, to address these potential biases. Third, we did not examine self-employed respondents because they lacked data on certain PE indicators and may have a different social class than non-self-employed respondents (eg, business owners versus workers). Therefore, these results are not generalizable to the 8% of the US population that is self-employed. Fourth, we relied on data from O*NET to operationalize interpersonal power relations; data from O*NET are time-fixed and only changed for an individual in our sample if they changed jobs during follow-up. This is likely why we did not see large increases in the dimension over time. Fifth, our carrying forwards and backwards of the promotion variable could have introduced measurement error in the training opportunities dimension in the early 1990s. Finally, although a multidimensional indicator has many strengths, it could mask heterogeneous trends within dimensions. Despite these limitations, the NLSY is one of the longest running studies in the US of a racially, ethnically and economically diverse sample of adults, spanning early adulthood through age 60, and thus, was the best individual-level longitudinal dataset available for our research question.

### Concluding remarks

This is the first study to describe trends in PE in the US using a multidimensional indicator and longitudinal measurement. We found that PE increased between 1988 and 2016 by 9% overall and with variation among all subgroups. The PES was persistently higher and increased over time among women and those from racialized and less-educated populations, but the largest increases were observed among male, college-educated and higher-income individuals. Future work should strongly consider multidimensional employment quality indicators and longitudinal measurement, given that these widespread increases in PE could deleteriously affect public health.

## Supplementary material

Supplementary material
