# Supplementary material for: Changes in precarious employment in the United States: A longitudinal analysis
Source: Scand J Work Environ Health. 2021 Mar 31;47(3):171–80. doi: 10.5271/sjweh.3939 (PMC8126438; doi:10.5271/sjweh.3939)
Supplement: Supplementary material [file SJWEH-47-171-S001.pdf]

Changes in precarious employment in the United States: A longitudinal analysis[1]

by Vanessa M Oddo, PhD,[2] Castiel Chen Zhuang, MS, Sarah B Andrea, PhD, Jerzy Eisenberg-Guyot, PhD, Trevor Peckham, PhD, Daniel Jacoby, PhD, Anjum Hajat, PhD

1. *Supplementary material*
2. *Correspondence to: Vanessa M Oddo, University of Illinois Chicago, Department of Kinesiology and Nutrition, 1919 West Taylor Street, MC 517, Chicago IL, 60612, USA. [E-mail: voddo@uic.edu]*

Supplemental Figure S1. Sample Distribution of Income from Wages/Salary

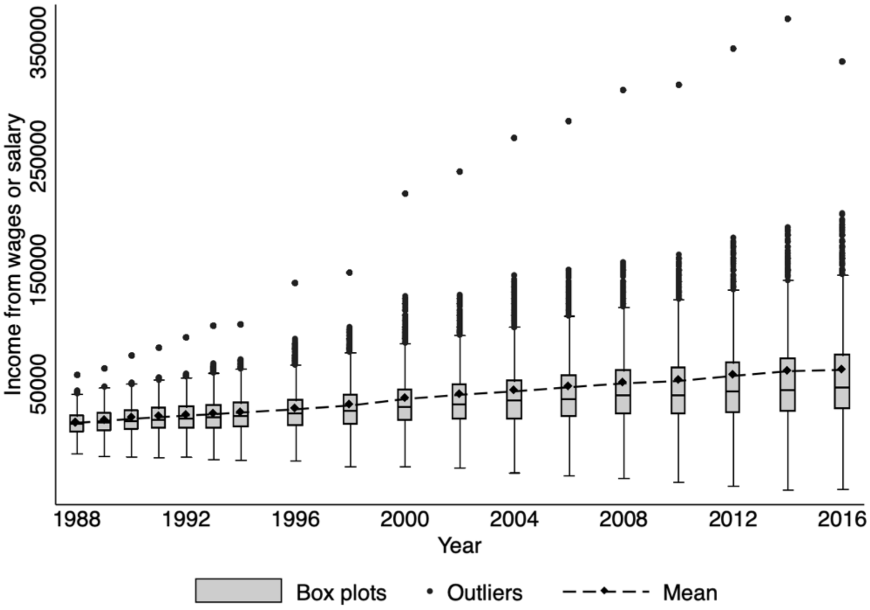

Supplemental Figure S2. Precarious Employment Score Sensitivity Analyses [a-e]

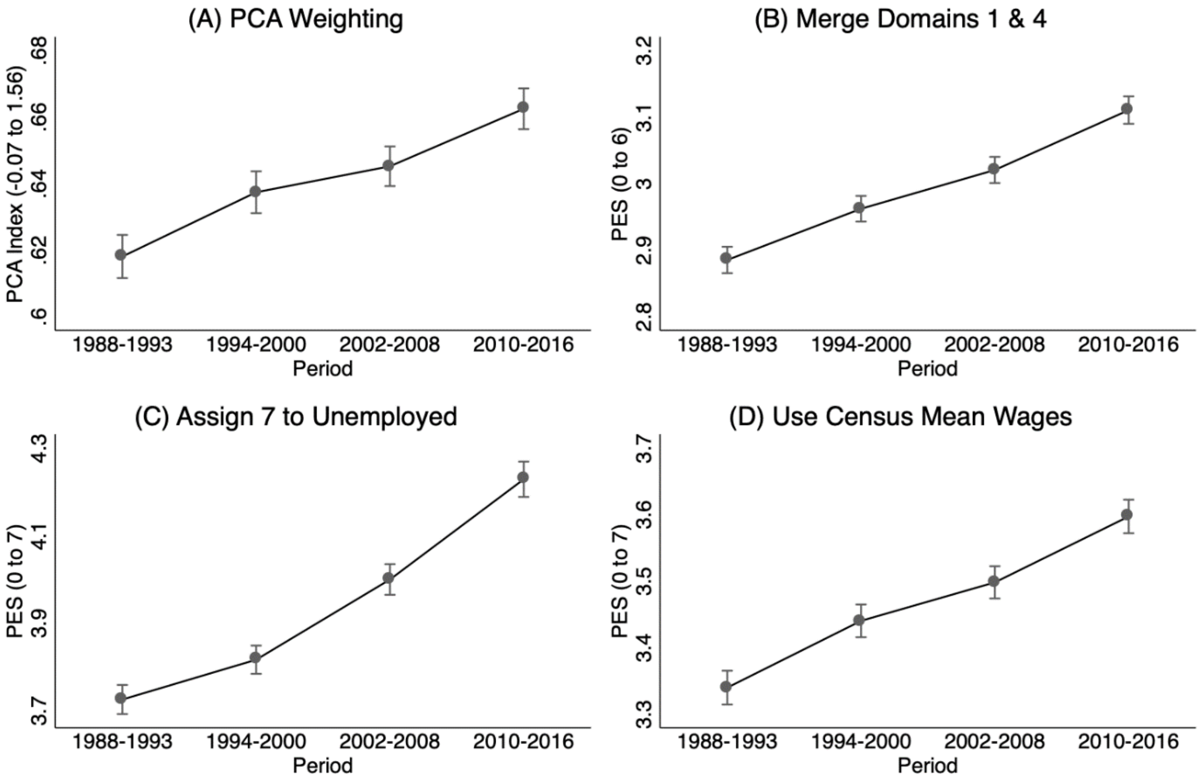

CI = confidence interval; PCA = principal components analysis; PES = precarious employment score

[a] We estimated the PES using a GEE model with an unstructured correlation structure and time period indicators, then predicted the averages at each time period with their 95% CIs.

[b] Panel A details the PCA-driven PES over time (range -0.07 – 1.56). We applied an oblique rotation to our loadings to allow for the theoretical and observed correlation between components. For each component, we calculated the linear sum of each standardized item value multiplied by their factor loading. The precarious employment score was equal to the linear sum of all components with each component weighted by the percentage of the variance it explained. The data-driven PES suggested a 5-domain solution, which we termed as follows based on the variables with highest loadings in each domain: 1) fringe benefits (paid vacation, health insurance, retirement plan, training opportunities); 2) traditional employment (wages, regular hours, promotion opportunities, freedom to make decisions); 3) instability (weeks employed and tenure in employment); 4) stability (fixed hours, union membership); 5) work schedule (regular shift/schedule).

[c] Panel B details the age-adjusted PES over time, when combing the material rewards and worker's rights domains (range 0 – 6).

[d] Panel C details the age-adjusted PES over time, when we include observations from unemployed individuals and assign them a 7 (range 0 – 7).

[e] Panel D details the age-adjusted PES over time, when we use the age-year specific census mean value to dichotomize lower- versus higher-income individuals (range 0 – 7).

Supplemental Figure S3. Precarious Employment Score Over Time with Categorical Indicators of the Survey Waves, 1988-2016[a,b]

(A) PES Over Time (B) Comparing the PES to 1988

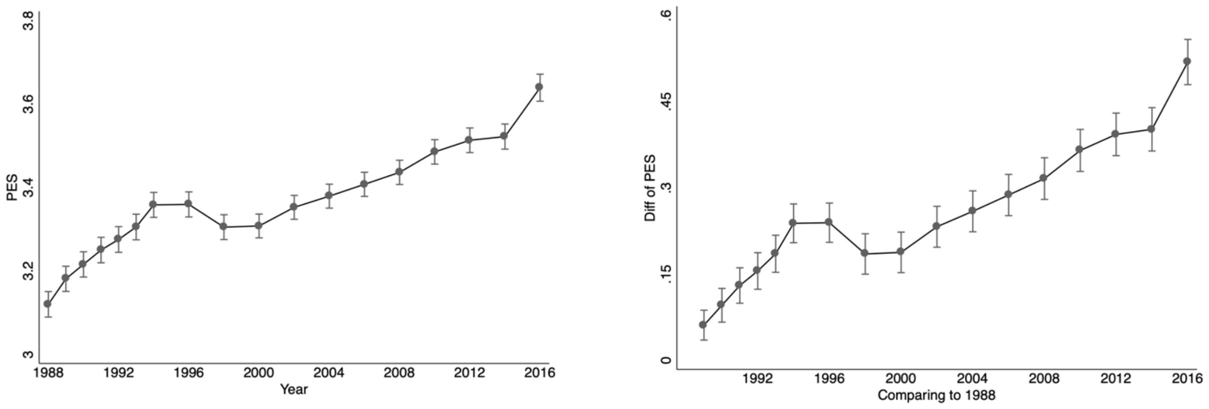

CI = confidence interval; PES = precarious employment score

[a] The PES is adjusted to age 30.

[b] We estimated the predicted precarious employment score using a GEE model with an unstructured correlation structure and wave fixed-effects. Panel A displays the average PES (95% CI) at each survey wave. Panel B compares the change in the average PES from 1988 to subsequent survey waves.

Supplemental Table S1. Precarious Employment Score at Age 30 by Year[a]

Year	Mean or Proportion (Standard Deviation)						
	PES	Middle Tertile[b]	Upper Tertile[b]	Females PES	Non-Hispanic Blacks PES	Primary School PES	South PES
1988	2.96 (1.23)	27% (0.45)	27% (0.45)	3.09 (1.22)	3.10 (1.27)	3.68 (1.23)	2.98 (1.19)
1989	3.00 (1.23)	27% (0.44)	28% (0.45)	3.18 (1.22)	3.12 (1.24)	3.69 (1.47)	3.05 (1.20)
1990	3.03 (1.23)	26% (0.44)	29% (0.45)	3.21 (1.24)	3.15 (1.28)	3.95 (1.04)	3.10 (1.19)
1991	3.05 (1.25)	25% (0.43)	29% (0.46)	3.22 (1.24)	3.19 (1.29)	3.90 (1.19)	3.14 (1.21)
1992	3.07 (1.27)	25% (0.43)	30% (0.46)	3.26 (1.26)	3.20 (1.29)	4.15 (1.16)	3.13 (1.20)
1993	3.11 (1.26)	26% (0.44)	30% (0.46)	3.28 (1.27)	3.21 (1.29)	3.79 (1.14)	3.15 (1.22)
1994	3.15 (1.21)	26% (0.44)	31% (0.46)	3.28 (1.22)	3.31 (1.24)	3.71 (1.11)	3.17 (1.19)
1996	3.20 (1.23)	28% (0.45)	32% (0.47)	3.36 (1.24)	3.37 (1.29)	3.98 (0.82)	3.26 (1.20)
1998	3.16 (1.21)	28% (0.45)	31% (0.46)	3.35 (1.22)	3.30 (1.24)	4.10 (1.13)	3.20 (1.18)
2000	3.17 (1.15)	30% (0.46)	30% (0.46)	3.35 (1.17)	3.34 (1.22)	3.96 (0.95)	3.24 (1.14)
2002	3.20 (1.14)	29% (0.45)	31% (0.46)	3.40 (1.15)	3.39 (1.18)	4.29 (0.88)	3.28 (1.13)
2004	3.21 (1.12)	29% (0.45)	32% (0.47)	3.39 (1.12)	3.37 (1.16)	4.15 (0.80)	3.28 (1.11)
2006	3.26 (1.13)	29% (0.46)	33% (0.47)	3.43 (1.13)	3.38 (1.14)	3.96 (1.02)	3.34 (1.12)
2008	3.28 (1.13)	30% (0.46)	34% (0.47)	3.44 (1.15)	3.39 (1.15)	4.12 (0.98)	3.35 (1.10)
2010	3.32 (1.11)	32% (0.47)	34% (0.47)	3.46 (1.13)	3.46 (1.16)	4.26 (0.91)	3.40 (1.10)
2012	3.32 (1.09)	31% (0.46)	33% (0.47)	3.48 (1.10)	3.48 (1.17)	4.19 (0.87)	3.39 (1.09)
2014	3.32 (1.08)	31% (0.46)	32% (0.47)	3.48 (1.12)	3.44 (1.15)	4.34 (0.94)	3.38 (1.09)
2016	3.43 (1.15)	30% (0.46)	35% (0.48)	3.59 (1.16)	3.55 (1.22)	4.30 (1.05)	3.49 (1.13)

PES = precarious employment score

[a] Ranges between 0 and 7. All numbers are weighted using the NLSY custom weights for the whole study period (1988-2016).

[b] Tertiles are created using data points in all years (N=101,290).

Supplemental Table S2. Precarious Employment Score Properties by Year[a]

Theory- versus data-driven scores:				
Year	Expected Agreement	Observed Agreement	Kappa Statistic	Spearman's correlation
1988	25%	61%	0.48	0.80
1989	25%	64%	0.52	0.82
1990	25%	65%	0.53	0.83
1991	25%	64%	0.52	0.84
1992	25%	64%	0.52	0.83
1993	25%	64%	0.52	0.83
1994	25%	63%	0.51	0.82
1996	25%	65%	0.53	0.83
1998	25%	64%	0.52	0.83
2000	25%	62%	0.50	0.81
2002	25%	62%	0.50	0.81
2004	25%	62%	0.49	0.81
2006	25%	62%	0.50	0.80
2008	25%	62%	0.49	0.81
2010	25%	61%	0.48	0.79
2012	25%	60%	0.47	0.79
2014	25%	60%	0.47	0.79
2016	25%	62%	0.49	0.80

[a]All correlations are significant at 1% level

Supplemental Table S3. Time Trend of the Precarious Employment Score when Including Unemployed Individuals [a]

	PES (95% CI)	Difference in Average PES (95% CI) [b]	Time x Subgroup (95% CI) [c]
**Overall Time Trend**			
1988-1993 (TP1)	3.73 (3.69, 3.76)	Reference	--
1994-2000 (TP2)	3.82 (3.78, 3.85)	0.09 (0.07, 0.12)	--
2002-2008 (TP3)	4.00 (3.96, 4.03)	0.27 (0.24, 0.31)	--
2010-2016 (TP4)	4.23 (4.19, 4.27)	0.50 (0.46, 0.54)	--
**Time Trend by Race/Ethnicity**			
1988-1993 (TP1)	3.73 (3.69, 3.76)	Reference	
1994-2000 (TP2)	3.82 (3.79, 3.85)	0.09 (0.07, 0.12)	
2002-2008 (TP3)	4.00 (3.97, 4.03)	0.27 (0.24, 0.31)	
2010-2016 (TP4)	4.23 (4.19, 4.27)	0.50 (0.46, 0.54)	
NH White	3.74 (3.70, 3.77)	Reference	--
Hispanic	4.01 (3.95, 4.08)	0.28 (0.20, 0.36)	--
NH Black	4.12 (4.07, 4.18)	0.39 (0.32, 0.45)	--
NH Others	4.08 (3.83, 4.34)	0.35 (0.09, 0.61)	--
TP1 NH White	3.58 (3.53, 3.62)	Reference	--
TP2 NH White	3.66 (3.61, 3.70)	0.08 (0.05, 0.12)	--
TP3 NH White	3.83 (3.78, 3.88)	0.25 (0.21, 0.30)	--
TP4 NH White	4.01 (3.96, 4.07)	0.44 (0.38, 0.49)	--
TP1 Hispanic	3.81 (3.73, 3.89)	Reference	Reference
TP2 Hispanic	3.94 (3.86, 4.01)	0.13 (0.07, 0.19)	0.05 (-0.02, 0.11)
TP3 Hispanic	4.13 (4.05, 4.21)	0.33 (0.25, 0.40)	0.07 (-0.02, 0.17)
TP4 Hispanic	4.34 (4.25, 4.44)	0.54 (0.44, 0.63)	0.10 (-0.01, 0.20)
TP1 NH Black	3.93 (3.86, 3.99)	Reference	Reference
TP2 NH Black	4.01 (3.95, 4.07)	0.08 (0.04, 0.13)	-0.00 (-0.06, 0.06)
TP3 NH Black	4.20 (4.14, 4.27)	0.28 (0.21, 0.34)	0.02 (-0.06, 0.11)
TP4 NH Black	4.52 (4.45, 4.60)	0.60 (0.52, 0.67)	0.16 (0.06, 0.25)
TP1 NH Others	3.98 (3.70, 4.27)	Reference	Reference
TP2 NH Others	3.98 (3.68, 4.28)	-0.00 (-0.21, 0.20)	-0.09 (-0.29, 0.12)
TP3 NH Others	4.22 (3.89, 4.54)	0.23 (-0.04, 0.51)	-0.02 (-0.30, 0.26)
TP4 NH Others	4.24 (3.89, 4.59)	0.25 (-0.09, 0.59)	-0.19 (-0.53, 0.16)
**Time Trend by Gender**			
1988-1993 (TP1)	3.73 (3.69, 3.76)	Reference	--
1994-2000 (TP2)	3.82 (3.79, 3.85)	0.09 (0.07, 0.12)	--
2002-2008 (TP3)	4.00 (3.97, 4.04)	0.28 (0.24, 0.31)	--
2010-2016 (TP4)	4.23 (4.19, 4.27)	0.50 (0.46, 0.54)	--

Male	3.70 (3.66, 3.74)	Reference	--
Female	4.12 (4.07, 4.16)	0.42 (0.36, 0.48)	--
TP1 Male	3.47 (3.42, 3.51)	Reference	--
TP2 Male	3.59 (3.55, 3.64)	0.12 (0.09, 0.16)	--
TP3 Male	3.83 (3.78, 3.88)	0.36 (0.31, 0.41)	--
TP4 Male	4.09 (4.03, 4.14)	0.62 (0.56, 0.67)	--
TP1 Female	3.98 (3.94, 4.03)	Reference	--
TP2 Female	4.04 (4.00, 4.09)	0.06 (0.02, 0.10)	-0.06 (-0.11, -0.02)
TP3 Female	4.18 (4.13, 4.23)	0.19 (0.14, 0.24)	-0.17 (-0.24, -0.10)
TP4 Female	4.37 (4.32, 4.43)	0.39 (0.33, 0.45)	-0.23 (-0.31, -0.15)
**Time Trend by Education Level**			
1988-1993 (TP1)	3.70 (3.67, 3.74)	Reference	--
1994-2000 (TP2)	3.81 (3.77, 3.84)	0.10 (0.08, 0.13)	--
2002-2008 (TP3)	4.01 (3.97, 4.04)	0.30 (0.27, 0.34)	--
2010-2016 (TP4)	4.28 (4.24, 4.32)	0.58 (0.54, 0.62)	--
Primary School	4.74 (4.43, 5.04)	Reference	--
High School	4.13 (4.09, 4.17)	-0.61 (-0.92, -0.30)	--
College	3.75 (3.71, 3.79)	-0.99 (-1.30, -0.67)	--
Graduate	3.30 (3.24, 3.37)	-1.43 (-1.75, -1.12)	--
TP1 Primary School	4.79 (4.43, 5.15)	Reference	--
TP2 Primary School	4.71 (4.34, 5.09)	-0.08 (-0.37, 0.21)	--
TP3 Primary School	4.81 (4.35, 5.27)	0.02 (-0.46, 0.50)	--
TP4 Primary School	4.59 (4.17, 5.01)	-0.20 (-0.69, 0.29)	--
TP1 High School	3.90 (3.86, 3.94)	Reference	--
TP2 High School	4.02 (3.98, 4.06)	0.12 (0.09, 0.15)	0.20 (-0.10, 0.49)
TP3 High School	4.21 (4.17, 4.26)	0.31 (0.27, 0.36)	0.30 (-0.19, 0.78)
TP4 High School	4.57 (4.51, 4.63)	0.67 (0.61, 0.73)	0.87 (0.37, 1.36)
TP1 College	3.54 (3.49, 3.59)	Reference	--
TP2 College	3.65 (3.61, 3.70)	0.11 (0.07, 0.15)	0.19 (-0.10, 0.48)
TP3 College	3.88 (3.83, 3.93)	0.34 (0.29, 0.40)	0.32 (-0.16, 0.81)
TP4 College	4.09 (4.04, 4.15)	0.55 (0.49, 0.61)	0.75 (0.25, 1.25)
TP1 Graduate	3.23 (3.14, 3.32)	Reference	--
TP2 Graduate	3.21 (3.12, 3.30)	-0.02 (-0.10, 0.06)	0.06 (-0.24, 0.36)
TP3 Graduate	3.36 (3.28, 3.45)	0.13 (0.03, 0.24)	0.12 (-0.37, 0.61)
TP4 Graduate	3.48 (3.39, 3.58)	0.26 (0.14, 0.37)	0.46 (-0.05, 0.96)

CI = confidence interval; NH = non-Hispanic; PES = precarious employment score; TP = time period; TP1 = 1988-1993; TP2 = 1994-2000; TP3 = 2002-2008; TP4 = 2010-2016

[a] Unemployed individuals are assigned a 7 (most precarious). The PES is adjusted to age 30. Estimates were calculated using separate GEE regression models with an unstructured

correlation structure. All models included categorical indicators of year (1988-1993, 1994-2000, 2002-2008, 2010-2016) and employed robust standard errors. Subgroup models include a time X subgroup interaction term.

[b] Compares within subgroup change over time. For example, it compares the PES for NH Whites in time period 1 to the PES for NH Whites in time period 2.

[c] Compares the between subgroup change over time. For example, the change in PES between time period 1 and time period 2, comparing NH Whites (0.08) and Hispanics (0.13), is 0.05.

Supplemental Table S4. Time Trend of the Precarious Employment Score when Using the Census Mean Income to Define Lower-and Higher-Income [a]

	PES (95% CI)	Difference in Average PES (95% CI) [b]	Time x Subgroup (95% CI) [c]
**Overall Time Trend**			
1988-1993 (TP1)	3.34 (3.31, 3.37)	Reference	--
1994-2000 (TP2)	3.44 (3.42, 3.46)	0.10 (0.08, 0.12)	--
2002-2008 (TP3)	3.50 (3.47, 3.52)	0.16 (0.13, 0.18)	--
2010-2016 (TP4)	3.60 (3.57, 3.62)	0.26 (0.23, 0.28)	--
**Time Trend by Race/Ethnicity**			
1988-1993 (TP1)	3.34 (3.31, 3.36)	Reference	
1994-2000 (TP2)	3.44 (3.41, 3.46)	0.10 (0.08, 0.12)	
2002-2008 (TP3)	3.49 (3.47, 3.52)	0.16 (0.13, 0.18)	
2010-2016 (TP4)	3.59 (3.57, 3.62)	0.26 (0.23, 0.28)	
NH White	3.35 (3.32, 3.38)	Reference	--
Hispanic	3.50 (3.45, 3.54)	0.14 (0.08, 0.20)	--
NH Black	3.56 (3.53, 3.60)	0.21 (0.16, 0.26)	--
NH Other	3.55 (3.36, 3.75)	0.13 (0.00, 0.40)	--
TP1 NH White	3.25 (3.22, 3.29)	Reference	--
TP2 NH White	3.34 (3.31, 3.37)	0.09 (0.06, 0.11)	--
TP3 NH White	3.41 (3.37, 3.44)	0.16 (0.12, 0.19)	--
TP4 NH White	3.51 (3.48, 3.54)	0.26 (0.22, 0.29)	--
TP1 Hispanic	3.37 (3.31, 3.43)	Reference	--
TP2 Hispanic	3.51 (3.46, 3.57)	0.15 (0.10, 0.19)	0.06 (0.01, 0.11)
TP3 Hispanic	3.57 (3.51, 3.63)	0.20 (0.15, 0.26)	0.05 (-0.02, 0.11)
TP4 Hispanic	3.64 (3.58, 3.70)	0.27 (0.21, 0.33)	0.02 (-0.05, 0.09)
TP1 NH Black	3.46 (3.42, 3.51)	Reference	--
TP2 NH Black	3.56 (3.51, 3.61)	0.10 (0.06, 0.13)	0.01 (-0.04, 0.06)
TP3 NH Black	3.61 (3.56, 3.65)	0.14 (0.10, 0.19)	-0.01 (-0.07, 0.04)
TP4 NH Black	3.72 (3.67, 3.77)	0.26 (0.21, 0.31)	-0.00 (-0.06, 0.06)
TP1 NH Other	3.59 (3.35, 3.82)	Reference	--
TP2 NH Other	3.60 (3.36, 3.83)	0.01 (-0.15, 0.16)	-0.08 (-0.24, 0.08)
TP3 NH Other	3.48 (3.27, 3.70)	-0.11 (-0.30, 0.09)	-0.26 (-0.46, -0.06)
TP4 NH Other	3.52 (3.30, 3.74)	-0.07 (-0.27, 0.13)	-0.33 (-0.53, -0.13)
**Time Trend by Gender**			
1988-1993 (TP1)	3.34 (3.31, 3.36)	Reference	
1994-2000 (TP2)	3.44 (3.41, 3.46)	0.10 (0.08, 0.12)	
2002-2008 (TP3)	3.49 (3.47, 3.52)	0.16 (0.13, 0.18)	
2010-2016 (TP4)	3.59 (3.57, 3.62)	0.26 (0.23, 0.28)	
Male	3.32 (3.29, 3.35)	Reference	--

Female	3.57 (3.54, 3.60)	0.25 (0.21, 0.29)	--
TP1 Male	3.19 (3.16, 3.23)	Reference	--
TP2 Male	3.32 (3.28, 3.35)	0.13 (0.10, 0.15)	--
TP3 Male	3.39 (3.35, 3.42)	0.19 (0.16, 0.23)	--
TP4 Male	3.51 (3.48, 3.55)	0.32 (0.28, 0.35)	--
TP1 Female	3.49 (3.46, 3.53)	Reference	--
TP2 Female	3.57 (3.53, 3.60)	0.07 (0.04, 0.10)	-0.05 (-0.09, -0.01)
TP3 Female	3.61 (3.58, 3.65)	0.12 (0.08, 0.15)	-0.07 (-0.12, -0.03)
TP4 Female	3.68 (3.65, 3.72)	0.19 (0.15, 0.23)	-0.13 (-0.18, -0.08)
**Time Trends by Educational Level**			
1988-1993 (TP1)	3.31 (3.28, 3.33)	Reference	--
1994-2000 (TP2)	3.42 (3.39, 3.44)	0.11 (0.09, 0.13)	--
2002-2008 (TP3)	3.50 (3.48, 3.52)	0.19 (0.16, 0.21)	--
2010-2016 (TP4)	3.63 (3.60, 3.65)	0.32 (0.29, 0.35)	--
Primary School	4.18 (3.96, 4.40)	Reference	--
High School	3.65 (3.62, 3.68)	-0.53 (-0.75, -0.30)	--
College	3.28 (3.25, 3.31)	-0.90 (-1.12, -0.67)	--
Graduate	2.95 (2.90, 3.00)	-1.23 (-1.45, -1.00)	--
TP1 Primary School	4.17 (3.86, 4.48)	Reference	--
TP2 Primary School	4.11 (3.85, 4.37)	-0.06 (-0.30, 0.17)	--
TP3 Primary School	4.17 (3.89, 4.46)	0.00 (-0.32, 0.32)	--
TP4 Primary School	4.30 (4.01, 4.58)	0.13 (-0.25, 0.51)	--
TP1 High School	3.51 (3.48, 3.55)	Reference	--
TP2 High School	3.66 (3.63, 3.69)	0.15 (0.12, 0.17)	0.21 (-0.03, 0.45)
TP3 High School	3.71 (3.68, 3.74)	0.20 (0.17, 0.23)	0.20 (-0.13, 0.52)
TP4 High School	3.84 (3.81, 3.88)	0.33 (0.29, 0.37)	0.20 (-0.18, 0.58)
TP1 College	3.14 (3.10, 3.18)	Reference	--
TP2 College	3.26 (3.22, 3.29)	0.11 (0.08, 0.15)	0.18 (-0.06, 0.41)
TP3 College	3.37 (3.34, 3.41)	0.23 (0.19, 0.27)	0.23 (-0.09, 0.55)
TP4 College	3.49 (3.46, 3.53)	0.35 (0.31, 0.39)	0.22 (-0.16, 0.60)
TP1 Graduate	2.95 (2.87, 3.02)	Reference	--
TP2 Graduate	2.85 (2.79, 2.91)	-0.09 (-0.16, -0.03)	-0.03 (-0.28, 0.21)
TP3 Graduate	2.94 (2.89, 2.99)	-0.01 (-0.08, 0.07)	-0.01 (-0.34, 0.32)
TP4 Graduate	3.11 (3.06, 3.16)	0.16 (0.08, 0.24)	0.04 (-0.35, 0.42)

CI = confidence interval; NH = non-Hispanic; PES = precarious employment score; TP = time period; TP1 = 1988-1993; TP2 = 1994-2000; TP3 = 2002-2008; TP4 = 2010-2016

[a] The PES is adjusted to age 30. Estimates were calculated using separate GEE regression models with an unstructured correlation structure. All models included categorical indicators of

year (1988-1993, 1994-2000, 2002-2008, 2010-2016) and employed robust standard errors. Subgroup models include a time X subgroup interaction term.
[b] Compares within subgroup change over time. For example, it compares the PES for NH Whites in time period 1 to the PES for NH Whites in time period 2.
[c] Compares the between subgroup change over time. For example, the change in PES between time period 1 and time period 2, comparing NH Whites (0.09) and Hispanics (0.15), is 0.06.

Supplemental Table S5. Year Trend of the PES Overall and by Subgroups [a]

	Average PES (95% CI)	Difference in Average PES (95% CI) [b]	Year x Subgroup (95% CI) [c]
**Overall Year Trend**			
1988	3.12 (3.09, 3.15)	Reference	--
2016	3.64 (3.61, 3.67)	0.52 (0.48, 0.56)	--
**Year Trend by Race/Ethnicity**			
1988 NH White	3.03 (2.99, 3.07)	Reference	--
2016 NH White	3.54 (3.50, 3.59)	0.52 (0.47, 0.57)	--
1988 Hispanic	3.17 (3.10, 3.24)	Reference	--
2016 Hispanic	3.73 (3.65, 3.81)	0.56 (0.47, 0.65)	0.04 (-0.06, 0.15)
1988 NH Black	3.23 (3.18, 3.29)	Reference	--
2016 NH Black	3.75 (3.68, 3.81)	0.51 (0.43, 0.59)	-0.01 (-0.10, 0.09)
1988 NH Others	3.48 (3.21, 3.76)	Reference	--
2016 NH Others	3.66 (3.33, 3.98)	0.17 (-0.22, 0.56)	-0.34 (-0.74, 0.05)
**Year Trend by Gender**			
1988 Male	2.98 (2.94, 3.02)	Reference	--
2016 Male	3.55 (3.50, 3.69)	0.57 (0.51, 0.62)	--
1988 Female	3.27 (3.22, 3.31)	Reference	--
2016 Female	3.73 (3.69, 3.78)	0.46 (0.41, 0.52)	-0.10 (-0.18, -0.03)
**Year Trend by Education Level**			
1988 Primary School	4.03 (3.67, 4.38)	Reference	--
2016 Primary School	4.28 (3.90, 4.65)	0.25 (-0.27, 0.77)	--
1988 High School	3.26 (3.22, 3.30)	Reference	--
2016 High School	3.93 (3.88, 3.98)	0.66 (0.60, 0.72)	0.41 (-0.11, 0.93)
1988 College	2.91 (2.86, 2.96)	Reference	--
2016 College	3.54 (3.49, 3.59)	0.63 (0.56, 0.69)	0.38 (-0.15, 0.90)
1988 Graduate	2.84 (2.73, 2.96)	Reference	--
2016 Graduate	3.09 (3.02, 3.16)	0.24 (0.12, 0.37)	-0.01 (-0.54, 0.53)
**Year Trends by Income**			
1988 Below Median	3.56 (3.52, 3.59)	Reference	--
2016 Below Median	3.97 (3.93, 4.01)	0.42 (0.37, 0.47)	--
1988 Above Median	2.58 (2.55, 2.62)	Reference	--
2016 Above Median	3.22 (3.18, 3.25)	0.64 (0.59, 0.68)	0.22 (0.15, 0.28)
**Year Trends by Region**			
1988 South	3.15 (3.11, 3.20)	Reference	--
2016 South	3.71 (3.66, 3.76)	0.56 (0.50, 0.62)	--
1988 Northeast	2.98 (2.91, 3.05)	Reference	--
2016 Northeast	3.49 (3.40, 3.57)	0.50 (0.40, 0.61)	-0.05 (-0.17, 0.06)
1988 North Central	3.13 (3.07, 3.19)	Reference	--

2016 North Central	3.66 (3.60, 3.72)	0.53 (0.45, 0.61)	-0.03 (-0.13, 0.07)
1988 West	3.15 (3.09, 3.22)	Reference	--
2016 West	3.57 (3.50, 3.64)	0.41 (0.32, 0.50)	-0.14 (-0.25, -0.04)

CI = confidence interval; NH = non-Hispanic; PES = precarious employment score

[a] The PES is adjusted to age 30. Estimates were calculated using 6 separate GEE regression models with an unstructured correlation structure. All models included year dummies and employed robust standard errors. Subgroup models include a year X subgroup interaction term. Results for intermediate years (1989-2014) are not reported.

[b] Compares within subgroup change over time. For example, it compares the PES for NH Whites in 1988 to the PES for NH Whites in 2016.

[c] Compares the between subgroup change over time. For example, the change in PES between 1988 and 2016, comparing NH Whites (0.52) and Hispanics (0.56), is 0.04.

Supplemental Table S6. Year Trend of the PES Overall [a]

	Average PES (95% CI)	Difference in Average PES (95% CI) [b]
**Overall Year Trend**		
1988	3.12 (3.09, 3.15)	Reference
1989	3.18 (3.15, 3.21)	0.06 (0.04, 0.09)
1990	3.22 (3.19, 3.25)	0.10 (0.07, 0.13)
1991	3.25 (3.22, 3.28)	0.13 (0.10, 0.16)
1992	3.28 (3.24, 3.31)	0.16 (0.12, 0.19)
1993	3.31 (3.27, 3.34)	0.19 (0.15, 0.22)
1994	3.36 (3.33, 3.39)	0.24 (0.20, 0.27)
1996	3.36 (3.33, 3.39)	0.24 (0.21, 0.27)
1998	3.30 (3.27, 3.33)	0.18 (0.15, 0.22)
2000	3.31 (3.28, 3.34)	0.19 (0.15, 0.22)
2002	3.35 (3.32, 3.38)	0.23 (0.20, 0.27)
2004	3.38 (3.35, 3.41)	0.26 (0.22, 0.29)
2006	3.41 (3.38, 3.44)	0.29 (0.25, 0.32)
2008	3.44 (3.41, 3.47)	0.32 (0.28, 0.35)
2010	3.48 (3.46, 3.51)	0.37 (0.33, 0.40)
2012	3.51 (3.48, 3.54)	0.39 (0.36, 0.43)
2014	3.52 (3.49, 3.55)	0.40 (0.36, 0.44)
2016	3.64 (3.61, 3.67)	0.52 (0.48, 0.56)

CI = confidence interval; PES = precarious employment score

[a] The PES is adjusted to age 30. Estimates were calculated using a GEE regression model with an unstructured correlation structure. The model included year dummies and employed robust standard errors.

[b] Compares change over time. For example, it compares the PES in 2016 (3.64) to the PES in 1988 (3.12).
